# Activation of TWIST Transcription by Chromatin Remodeling Protein BRG1 Contributes to Liver Fibrosis in Mice

**DOI:** 10.3389/fcell.2020.00340

**Published:** 2020-05-13

**Authors:** Wenhui Dong, Ming Kong, Yuwen Zhu, Yang Shao, Dongmei Wu, Jun Lu, Junli Guo, Yong Xu

**Affiliations:** ^1^Key Laboratory of Biotechnology on Medical Plants of Jiangsu Province and School of Life Sciences, Jiangsu Normal University, Xuzhou, China; ^2^Key Laboratory of Targeted Intervention of Cardiovascular Disease, Collaborative Innovation Center for Cardiovascular Translational Medicine, Department of Pathophysiology, Nanjing Medical University, Nanjing, China; ^3^Institute of Biomedical Research, Liaocheng University, Liaocheng, China; ^4^Hainan Provincial Key Laboratory for Tropical Cardiovascular Diseases Research and Key Laboratory of Emergency and Trauma of Ministry of Education, Institute of Cardiovascular Research of the First Affiliated Hospital, Hainan Medical University, Haikou, China

**Keywords:** transcriptional regulation, Brg1, epigenetics, liver fibrosis, endothelial cell

## Abstract

Liver fibrosis is a complex pathophysiological process to which many different cell types contribute. Endothelial cells play versatile roles in the regulation of liver fibrosis. The underlying epigenetic mechanism is not fully appreciated. In the present study, we investigated the role of BRG1, a chromatin remodeling protein, in the modulation of endothelial cells in response to pro-fibrogenic stimuli *in vitro* and liver fibrosis in mice. We report that depletion of BRG1 by siRNA abrogated TGF-β or hypoxia induced down-regulation of endothelial marker genes and up-regulation of mesenchymal marker genes in cultured endothelial cells. Importantly, endothelial-specific BRG1 deletion attenuated CCl_4_ induced liver fibrosis in mice. BRG1 knockdown *in vitro* or BRG1 knockout *in vivo* was accompanied by the down-regulation of TWIST, a key regulator of endothelial phenotype. Mechanistically, BRG1 interacted with and was recruited to the TWIST promoter by HIF-1α to activate TWIST transcription. BRG1 silencing rendered a more repressive chromatin structure surrounding the TWIST promoter likely contributing to TWIST down-regulation. Inhibition of HIF-1α activity dampened liver fibrosis in mice. Similarly, pharmaceutical inhibition of TWIST alleviated liver fibrosis in mice. In conclusion, our data suggest that epigenetic activation of TWIST by BRG1 contributes to the modulation of endothelial phenotype and liver fibrosis. Therefore, targeting the HIF1α-BRG1-TWIST axis may yield novel therapeutic solutions to treat liver fibrosis.

## Introduction

The liver is constantly exposed to a myriad of injurious stimuli, including pathogens, toxins, nutrients, and metabolites, which inevitably trigger the host defense mechanism (Shackel and Rockey, 2005). Fibrogenesis is part of the evolutionarily conserved host defense/wound healing process. On the one hand, fibrogenesis helps wound closure and preserve structural integrity of injured tissues and organs. Resolution of fibrogenesis leaves the hepatic anatomy intact and its key functions undisturbed. Excessive or uncontrolled fibrogenesis, however, serves to disrupt hepatic structures, severely compromises hepatic functions, and is often associated with end-stage liver diseases such as hepatocellular carcinoma and cirrhosis (Brenner et al., 2013). Irrespective of the etiologies, liver fibrogenesis is mediated by activated myofibroblasts capable of both muscle-like contraction and producing fibrillar proteins to remodel the extracellular matrix (Kisseleva, 2017).

Extensive research efforts have been invested in determining the source(s) of ECM-producing myofibroblasts during liver fibrosis. Initially most in the field thought hepatic stellate cells (HSCs) represent the predominant source of myofibroblasts in the liver regardless of etiologies (Brenner et al., 2000). Later it was suggested that other cell types, including portal fibroblast (Iwaisako et al., 2014), hepatocyte (Zeisberg et al., 2007), and cholangiocyte (Glaser et al., 2009), may contribute to the pool of activated myofibroblasts and consequently liver fibrosis. As the genetic lineage tracing technique becomes more sophisticated and more specific, HSCs once again have been shown to be the predominant source where activated myofibroblasts originate (Mederacke et al., 2013). Recently, Ribera et al. (2017), with the help of the same fate-mapping technique, have demonstrated that a small fraction of activated myofibroblasts in fibrotic livers could be traced back to endothelial cells likely via a process known as endothelial-to-mesenchymal transition (EndMT). Thus far, the notion that EndMT is a relevant process in liver fibrosis has been largely based on cell culture data *in vitro* whereas a recent single-cell RNA-seq (scRNA-seq) experiment aimed at delineating the identities of myofibroblasts in the fibrotic liver reveals that EndMT is unlikely to play a significant role in the pathogenesis of liver fibrosis *in vivo* (Dobie et al., 2019). From a pure transcriptional perspective, EndMT can be said to reflect a shift in gene expression patterns characterized by down-regulation of endothelial marker genes and up-regulation of mesenchymal marker genes. EndMT can be stimulated by a range of pathogenic factors, including TGF-β (Cooley et al., 2014), hypoxia (Xu et al., 2015), and IL-1β (Maleszewska et al., 2013). The epigenetic mechanism whereby the alterations of gene expression are regulated is not fully understood.

Brahma related gene 1 (BRG1) is the catalytic subunit of the mammalian chromatin remodeling complex. Accumulating evidence points to a pivotal role for BRG1 as a link between epigenetic regulation of transcription in endothelial cells and the pathogenesis of human diseases. For instance, Weng et al. (2015) have demonstrated that BRG1 activates the synthesis of endothelin (ET-1) in endothelial cells, which in turn promotes cardiac hypertrophy via paracrine/endocrine pathways. More recently, Zhang et al. (2018b) have reported that endothelial-derived, BRG1-dependent production of colony stimulating factor (CSF1) is responsible for macrophage trafficking and consequently abdominal aortic aneurysm. We have previously shown that endothelial-specific deletion of BRG1 in mice attenuates bile duct ligation (BDL) and thioacetamide induced liver fibrosis by regulating the transcription of caveolin-1 (CAV1) (Shao et al., 2020) and NADPH oxidase 4 (NOX4) (Li Z. et al., 2019), respectively. We report here that BRG1 is essential for EndMT in cultured cells and that endothelial-specific BRG1 deficiency attenuates carbon tetrachloride (CCl_4_) induced liver fibrosis in mice. Mechanistically, BRG1 epigenetically activates the transcription of TWIST, a key regulator of EndMT. Therefore, targeting the HIF1α-BRG1-TWIST axis may yield novel therapeutic solutions to treat liver fibrosis.

## Methods

### Animals

All animal experiments were reviewed and approved by the intramural Nanjing Medical University Ethics Committee on Humane Treatment of Experimental Animals. All mice were bred at the Nanjing Biomedical Research Institute of Nanjing University (NBRI). Endothelial-specific deletion of BRG1 was achieved by crossing the *Smarca4*^f/f^ strain (Li et al., 2018b) to the *Cdh5*-Cre^ERT2^ strain (Li et al., 2018c). To delete BRG1, 6-week male *Smarca4*^f/f^*; Cdh5*-Cre^ERT2^ mice were injected with Tamoxifen (1mg/kg) daily for 5 consecutive days; the age- and sex-matched control mice (*Smarca4*^f/f^) received the same injection regimen. Liver fibrosis was induced in mice by CCl_4_ injection (1.0 mL/kg body weight as 50%, vol/vol, weekly for 6 weeks) as previously described (Fan et al., 2015; Tian et al., 2015). The HIF1α inhibitors (LW-6, 20 mg/kg; YC-1, 30 mg/kg) were injected peritoneally every other day. The TWIST inhibitor (Harmine, 10 mg/kg) was injected peritoneally every other day.

### Cell Culture, Plasmids, Transient Transfection, and Reporter Assay

Human umbilical vascular endothelial cells (HUVEC, Lonza) were maintained in EGM-2media with supplements supplied by the vendor; three different batches of primary cells were used in this study. HEK293 cells and human immortalized umbilical endothelial cells (EAhy926) were maintained in DMEM supplemented with 10% FBS. Mouse primary liver sinusoidal endothelial cells (LSECs) were isolated and cultured as previously described (Meyer et al., 2016). Briefly, mice were anesthetized with isoflurane. Following perfusion and digestion, the liver suspension was passed through a 70 μm cell strainer. The non-parenchymal cells were isolated by density gradient centrifugation. LSECs were further purified by selective adherence for exactly 8 min. Purity of the isolated LSECs was verified by immunofluorescence staining with an anti-CD31 antibody (Abcam, ab28364). Typically, 95% of the isolated LSECs using this protocol stained positive for CD31. Primary hepatocytes (Li N. et al., 2018a) and primary HSCs (Kong et al., 2019) were isolated as previously described. BRG1 expression constructs (Li N. et al., 2018a) and TWIST promoter-luciferase constructs (Yang et al., 2008) have been previously described. Sequences for small interfering RNAs: siBRG1#1, AACATGCACCAGATGCACAAG; siBRG1#2, GCCCATGGAGTCCATGCAT; siHIF1, CUGAUGACCAGCAACUUGA. Transient transfection was performed with Lipofectamine 2000. Cells were harvested 48 h after transfection and reporter activity was measured using a luciferase reporter assay system (Promega).

### Protein Extraction and Western Blot

Whole cell lysates were obtained by re-suspending cell pellets in RIPA buffer (50 mM Tris pH7.4, 150 mM NaCl, 1% Triton X-100) with freshly added protease inhibitor (Roche) as previously described (Liu et al., 2018). Western blot analyses were performed with anti-BRG1 (Santa Cruz, sc-10768), anti-collagen type I (Rockland, 600-401-103), anti-α-SMA (Sigma, A2547), anti-HIF1α (Santa Cruz, sc-10790), anti-TWIST (Abcam, ab50887), anti-VE-Cadherin (Cell Signaling Technology, 2158), anti-PECAM1 (Proteintech, 11265-1), and anti-β-actin (Sigma, A2228) antibodies. For densitometrical quantification, densities of target proteins were normalized to those of β-actin. Data are expressed as relative protein levels compared to the control group which is arbitrarily set as 1.

### RNA Isolation and Real-Time PCR

RNA was extracted with the RNeasy RNA isolation kit (Qiagen). Reverse transcriptase reactions were performed using a SuperScript First-strand Synthesis System (Invitrogen). Real-time PCR reactions were performed on an ABI Prism 7500 system with the following primers: human PECAM1, 5′-CTGCTGACCCTTCTGCTCTGTTC-3′ and 5′-GGCAGGCTCT TCATGTCAACACT-3′; human CDH5, 5′-TCACCTTCT GCGAGGATATGG-3′ and 5′-GAGTTGAGCACCGACACATC-3′; human COL1A2, 5′-GTGGCAGTGATGGAAGTGTG-3′ and 5′-AGGACCAGCGTTACCAACAG-3′; human ACTA2, 5′-TCAATGTCCCAGCCATGTAT-3′ and 5′-CAGCACGATGC CAGTTGT-3′; human BRG1, 5′-TCATGTTGGCGAGCTA TTTCC-3′ and 5′-GGTTCCGAAGTCTCAACGATG-3′; human TWIST, 5′-GGCCGGAGACCTAGATG-3′ and 5′-ACGGGCCT GTCTCGCTTTCT-3′; mouse Pecam1, 5′-GACTCACGCTGG TGCTCTATGC-3′ and 5′-TCAGTTGCTGCCCATTCTCA-3′; mouse Cdh5, 5′-TCAACGCATCTGTGCCAGAGAT-3′ and 5′-CACGATTTGGTACAAGACAGTG-3′; mouse Col1a2, 5′-GC CACCATTGATAGTCTCTCC-3′ and 5′-CACCCCAGCGAAG AACTCATA-3′; mouse Acta2, 5′-ATAACCCTTCAGCGTTCA GCC-3′ and 5′-CCAACCATTACTCCCTGATGTCTG-3′; mouse Brg1, 5′-CAGTGGCTCAAGGCTATCG-3′ and 5′-TGTCTCG CTTACGCTTACG-3′; mouse Twist, 5′-GGACAAGCTGAGC AAGATTCA-3′ and 5′-CGGAGAAGGCGTAGCTGAG-3′. Ct values of target genes were normalized to the Ct values of housekeekping control gene (18s, 5′-CGCGGT TCTATTTTGTTGGT-3′ and 5′-TCGTCTTCGAAACTCCGA CT-3′ for both human and mouse genes) using the ΔΔCt method and expressed as relative mRNA expression levels compared to the control group which is arbitrarily set as 1.

### Chromatin Immunoprecipitation (ChIP)

Chromatin Immunoprecipitation (ChIP) assays were performed essentially as described before (Li Z. et al., 2019). In brief, chromatin in control and treated cells were cross-linked with 1% formaldehyde. Cells were incubated in lysis buffer (150 mM NaCl, 25 mM Tris pH 7.5, 1% Triton X-100, 0.1% SDS, 0.5% deoxycholate) supplemented with protease inhibitor tablet and PMSF. DNA was fragmented into ∼200 bp pieces using a Branson 250 sonicator. Aliquots of lysates containing 200 μg of protein were used for each immunoprecipitation reaction with anti-BRG1 (Santa Cruz, sc-10768), anti-HIF1α (Santa Cruz, sc-10790), anti-TWIST (Abcam, ab50887), anti-p300 (Santa Cruz, sc-585), anti-KMT2F/SETD1A (Bethyl Laboratories, A300-489A), anti-KDM3A/JMJD1A (Bethyl Laboratories, A301-538), anti-anti-acetyl H3 (Millipore, 06-599), anti-trimethyl H3K4 (Millipore, 07-449), anti-dimethylH3K9 (Millipore, 07-441), or pre-immune IgG. For re-ChIP, immune complexes were eluted with the elution buffer (1% SDS, 100 mM NaCO3), diluted with the re-ChIP buffer (1% Triton X-100, 2 mM EDTA, 150 mM NaCl, 20 mM Tris pH 8.1), and subject to immunoprecipitation with a second antibody of interest.

### Histology

Histologic analyses were performed essentially as described before. Briefly, paraffin-embedded sections were stained with picrosirius red (Sigma-Aldrich) or Masson’s trichrome (Sigma-Aldrich) according to standard procedures. Pictures were taken using an Olympus IX-70 microscope (Olympus, Tokyo, Japan). Quantifications were performed with Image J by two independent assessors. For each animal, at least three slides with ∼5 fields for each slide were included for quantification.

### Statistical Analysis

One-way ANOVA with *post-hoc* Scheffe analyses were performed by SPSS software (IBM SPSS v18.0, Chicago, IL, United States). Unless otherwise specified, values of *p <* 0.05 were considered statistically significant.

## Results

### BRG1 Is Essential for Endothelial-Mesenchymal Transition *in vitro*

**FIGURE 1:**
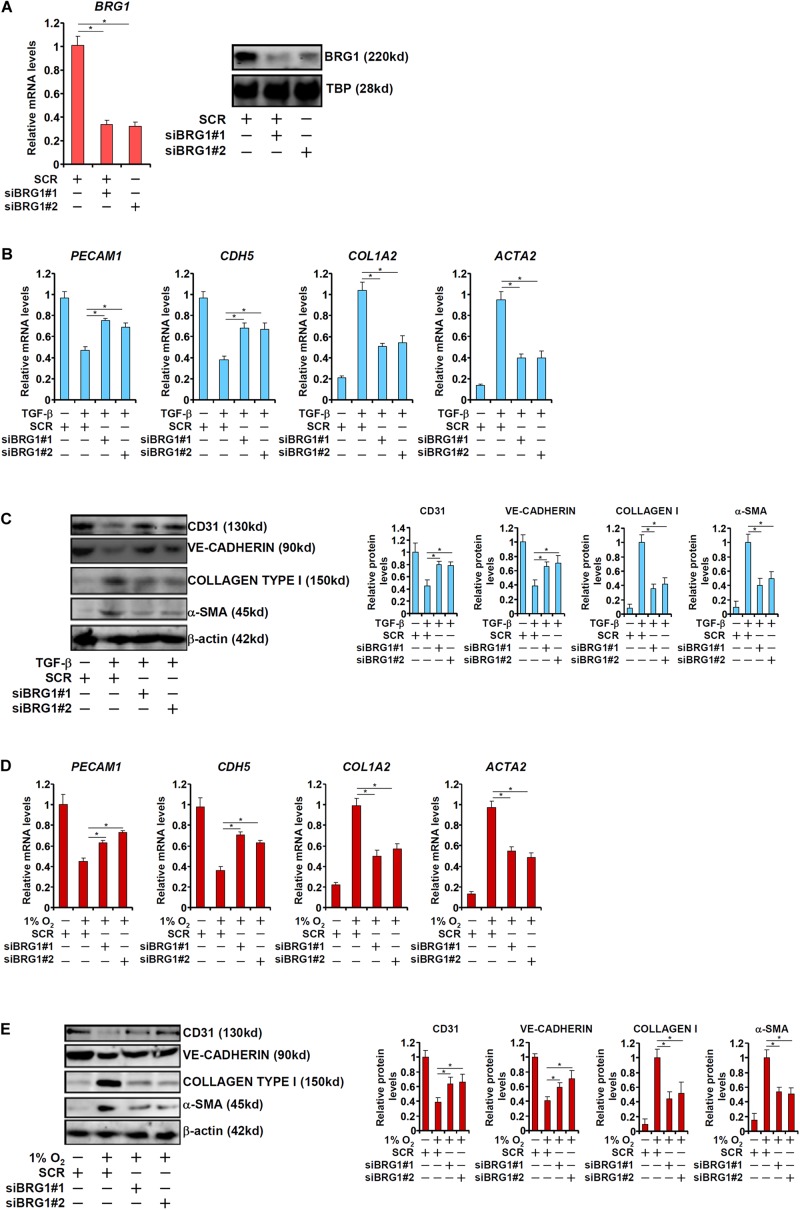
BRG1 is essential for endothelial-mesenchymal transition *in vitro*. **(A)** HUVECs were transfected with siRNA targeting BRG1 (siBRG1) or scrambled siRNA (SCR). BRG1 expression was examined by qPCR and Western. **(B,C)** HUVECs were transfected with siRNA targeting BRG1 or scrambled siRNA (SCR) followed by treatment with TGF-β. Gene expression levels were examined by qPCR and Western. **(D,E)** HUVECs were transfected with siRNA targeting BRG1 or scrambled siRNA (SCR) followed by treatment with hypoxia. Gene expression levels were examined by qPCR and Western. Error bars represent SEM (*p < 0.05, two-way ANOVA with *post-hoc* Scheffe test). All experiments were repeated three times and data represent averages of three independent experiments.

We first sought to determine the role of BRG1 in EndMT *in vitro* by treating primary human vascular endothelial cells with TGF-β, a prominent inducer of EndMT and fibrosis (Kovacic et al., 2012). TGF-β treatment potently down-regulated the expression of CD31 (*PECAM1*) and VE-Cadherin (*CDH5*) while at the same time up-regulating the expression of Collagen type I (*COL1A2*) and α-SMA (*ACTA2*). BRG1 knockdown by two separate pairs of siRNAs (Figure 1A for knockdown efficiencies), however, abolished TGF-β induced EndMT by increasing endothelial marker gene expression and decreasing mesenchymal marker gene expression at both mRNA (Figure 1B) and protein (Figure 1C) levels. We next exploited an alternative cell model of EndMT induced by hypoxia (1% O_2_), which has also been demonstrated to be a triggering factor for liver fibrosis (Tanne et al., 2005). As shown in Figures 1D,E, hypoxia stimulation similarly led to the down-regulation of *PECAM1* and *CDH5* and the up-regulation of *ACTA2* and *COL1A2* in endothelial cells, both of which were pre-empted by BRG1 silencing.

### Endothelial BRG1 Deficiency Attenuates Liver Fibrosis in Mice

We then made an attempt to authenticate the role of endothelial BRG1 in liver fibrosis. To this end, the *Smarca4*^f/f^ mice were crossbred to the *Cdh5*-Cre^ERT2^ mice to generate the endothelial conditional BRG1 knockout mice (ecKO); BRG1 deletion was achieved by Tamoxifen injection induced Cre expression and subsequent removal of the floxed *Smarca4* alleles. To verify the specificity and efficiency of BRG1 deletion, primary LSECs, hepatocytes, and HSCs were isolated from the WT and ecKO mice. Quantitative PCR analyses revealed that BRG1 expression was down-regulated by more than 60% in the LSECs isolated from the ecKO mice compared to those isolated from the WT mice. On the contrary, BRG1 expression was comparable in the hepatocytes and in the HSCs isolated from the WT mice and ecKO mice (Supplementary Figure S1).

**FIGURE 2:**
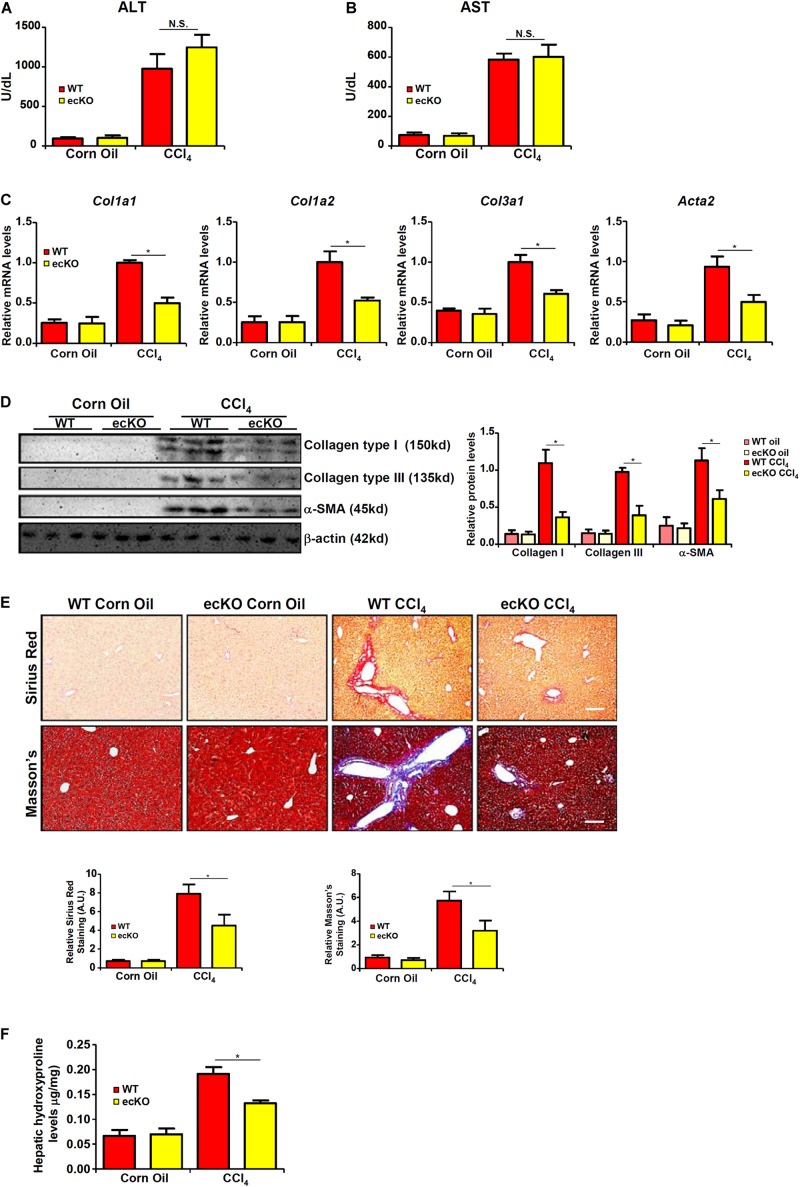
Endothelial BRG1 deficiency attenuates liver fibrosis in mice. Liver fibrosis was induced in endothelial-specific BRG1 knockout mice (ecKO) and wild type (WT) mice by CCl_4_ injection. **(A)** Plasma ALT levels. **(B)** Plasma AST levels. **(C,D)** Expression levels of pro-fibrogenic genes were examined by qPCR and Western. **(E)** Paraffin sections were stained with picrosirius red and Masson’s trichrome. **(F)** Hepatic hydroxylproline levels. *N* = 3∼4 mice for the corn oil groups and *N* = 6∼8 mice for the CCl_4_ groups. Error bars represent SD (*p < 0.05, two-way ANOVA with *post-hoc* Scheffe test).

WT and ecKO mice were subjected to chronic CCl_4_ injection to induce liver fibrosis. BRG1 ecKO and WT mice displayed comparable liver injury as measured by plasma ALT (Figure 2A) and AST (Figure 2B) levels. Quantitative PCR (Figure 2C) and Western blotting (Figure 2D) analyses showed that expression levels of pro-fibrogenic genes including collagen type I, collagen type III, and α-SMA were collectively down-regulated in ecKO mice compared to WT mice. Further, picrosirius red staining and Masson’s trichrome staining showed less extensive fibrosis in the ecKO livers than the WT livers (Figure 2E). Hepatic hydroxylproline quantification provided additional evidence that endothelial BRG1 deficiency attenuated liver fibrosis in mice (Figure 2F).

### BRG1 Interacts With HIF-1α to Regulate TWIST Transcription

**FIGURE 3:**
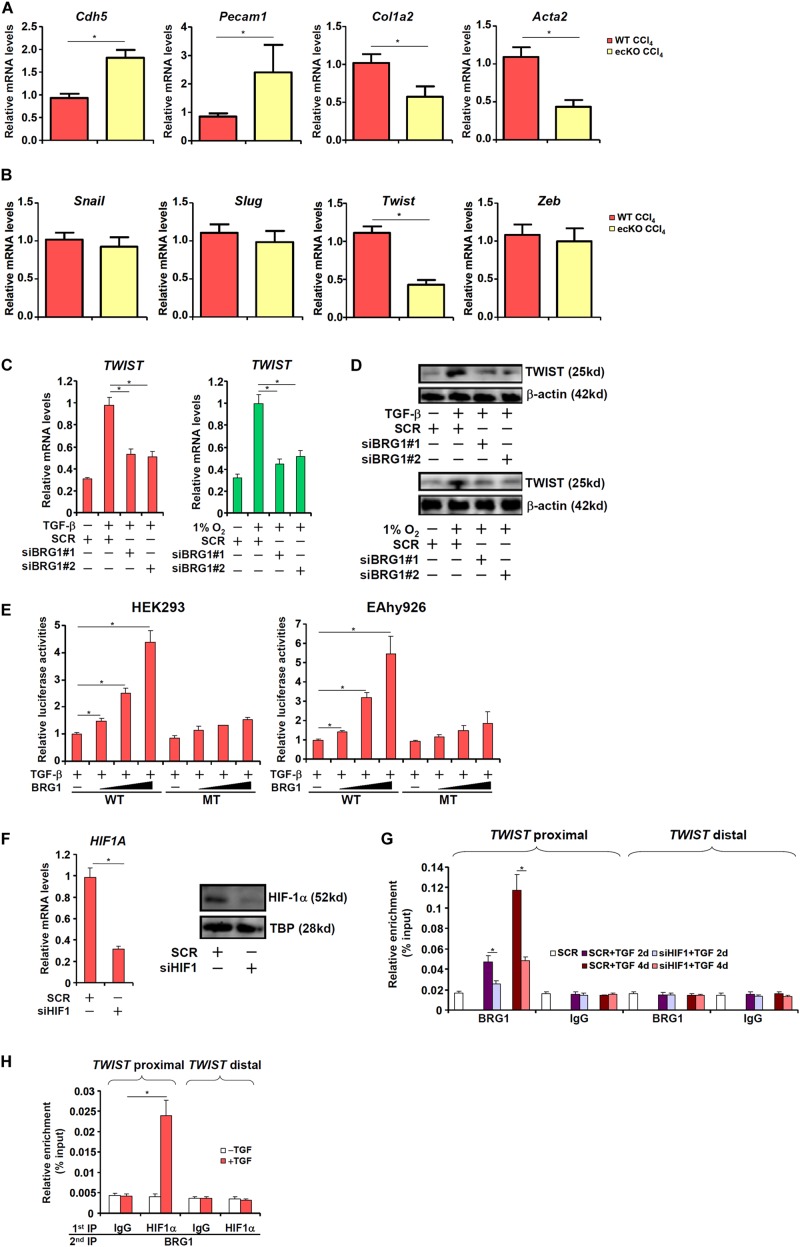
BRG1 regulates TWIST transcription. **(A,B)** Liver fibrosis was induced in WT and ecKO mice by CCl_4_ injection. Primary liver sinusoidal endothelial cells were isolated and gene expression levels were examined by qPCR. *N* = 6 mice for each group. **(C,D)** Human primary vascular endothelial cells were transfected with siRNA targeting BRG1 or SCR followed by treatment with TGF-β or hypoxia. TWIST expression was examined by qPCR and Western. **(E)** Wild type and HIF-1α mutant TWIST promoter-luciferase constructs were transfected into HEK293 cells or EAhy926 cells with or without BRG1. Luciferase activities were normalized by both protein concentration and GFP fluorescence. **(F,G)** Human primary vascular endothelial cells were transfected with siRNA targeting HIF-1α or SCR followed by treatment with TGF-β. HIF-1α expression was examined by qPCR and Western. ChIP assays were performed with indicated antibodies. **(H)** Human primary vascular endothelial cells were treated with or without TGF-β. Re-ChIP assays were performed with indicated antibodies. Error bars represent SEM (*p < 0.05, two-way ANOVA with *post-hoc* Scheffe test). All experiments were repeated three times and data represent averages of three independent experiments.

Of note, it was found that primary LSECs isolated from fibrotic ecKO mice receiving CCl_4_ injection expressed higher levels of endothelial marker genes (*Cdh5* and *Pecam1*) but lower levels of mesenchymal marker genes (*Col1a2* and *Acta2*), suggesting that BRG1 might regulate liver fibrosis by promoting EndMT *in vivo* (Figure 3A). The Snail family of zinc finger transcriptional factors, including Snail, Slug, Twist, and Zeb, specialize in repressing endothelial marker genes and are considered critical in EndMT (Wang et al., 2013b). As shown in Figure 3B, BRG1 deletion resulted in down-regulation of Twist but none of the other family members in LSECs (Figure 3B). We therefore hypothesized that BRG1 might regulate EndMT by activating Twist transcription. Depletion of BRG1 in human vascular endothelial cells dampened the induction of TWIST expression by both TGF-β treatment and hypoxia (Figures 3C,D). Moreover, co-transfection of BRG1 dose-dependently enhanced the activation of the TWIST promoter by TGF-β (Figure 3E). Interestingly, mutation of a binding site for HIF-1α within the proximal TWIST promoter abolished the trans-activation by BRG1 (Figure 3E), suggesting that HIF-1α might be responsible for recruiting BRG1 to the TWIST promoter. Indeed, ChIP assays showed that when the endothelial cells were exposed to TGF-β, BRG1 occupancies on the proximal TWIST promoter, but not the distal TWIST promoter, was significantly increased (Figure 3G); HIF-1α knockdown by siRNA (Figure 3F for knockdown efficiency) diminished the recruitment of BRG1. Finally, Re-ChIP assay demonstrated that BRG1 interacted with HIF-1α on the TWIST promoter in response to TGF-β (Figure 3H). Together, these data suggest that BRG1 might contribute to TWIST transcription in endothelial cells by interacting with HIF-1α.

### BRG1 Regulates TWIST Transcription by Modulating Histone Modifications

**FIGURE 4:**
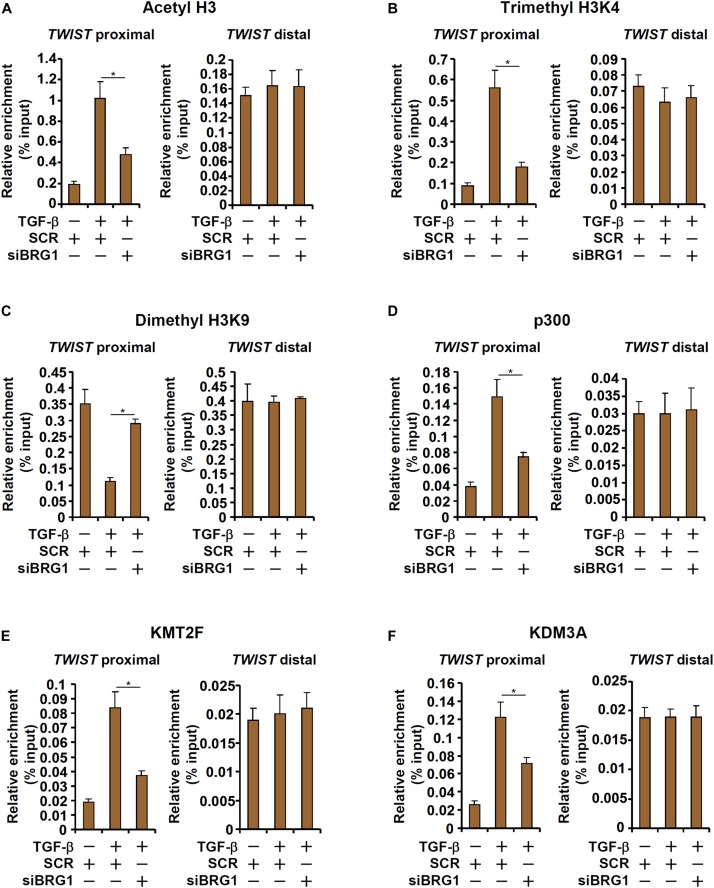
BRG1 regulates TWIST transcription by modulating histone modifications. **(A–F)** HUVECs were transfected with siRNA targeting BRG1 or SCR followed by treatment with TGF-β. ChIP assays were performed with anti-acetyl H3 **(A)**, anti-trimethyl H3K4 **(B)**, anti-dimethyl H3K9 **(C)**, anti-p300 **(D)**, anti-KMT2F **(E)**, and anti-KDM3A **(F)**. Error bars represent SEM (*p < 0.05, two-way ANOVA with *post-hoc* Scheffe test). All experiments were repeated three times and data represent averages of three independent experiments.

We next explored the epigenetic mechanism whereby BRG1 regulates TWIST transcription. Upon TGF-β stimulation, there was a simultaneous accumulation of acetylated H3 (AcH3, Figure 4A) and trimethylated H3K4 (H3K4Me3, Figure 4B), two signature active histone modifications, surrounding the proximal but not the distal TWIST promoter. On the contrary, dimethylated H3K9 (H3K9Me2, Figure 4C), a marker for repressed chromatin, was removed from the same region. BRG1 depletion by siRNA, however, significantly dampened the enrichment of AcH3 and H3K4Me3 and restored H3K9Me2 on the TWIST promoter. In accordance, it was observed that TGF-β stimulation provoked robust bindings of p300 (Figure 4D), a histone H3 acetyltransferase, KMT2F/SETD1A (Figure 4E), a histone H3K4 trimethyltransferase, and KDM3A/JMJD1A (Figure 4F), a histone H3K9 didemethylase, to the proximal TWIST promoter. BRG1 knockdown universally suppressed the recruitment of these histone modifying enzymes. Collectively, these data suggest that TWIST1 activation may be attributable to a dynamic interaction between BRG1 and various histone modifying enzymes.

### HIF-1α Inhibition Attenuates Liver Fibrosis in Mice

**FIGURE 5:**
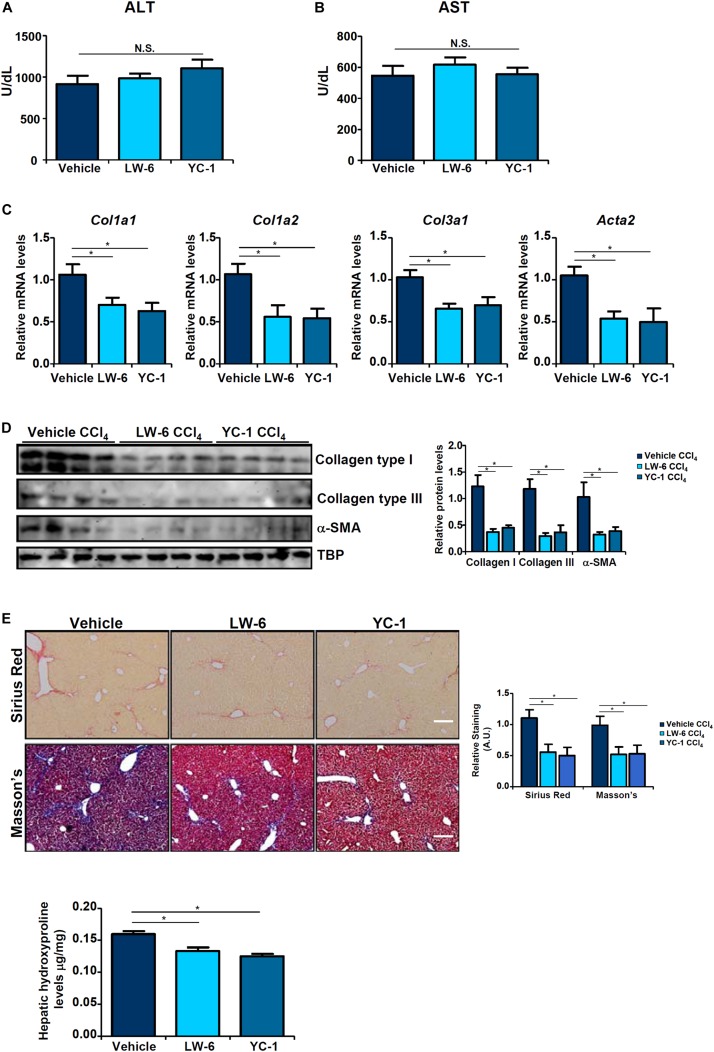
HIF-1α inhibition attenuates liver fibrosis in mice. Liver fibrosis was induced in by CCl_4_ injection. The mice were injected with two different HIF-1α inhibitors every other day. **(A)** Plasma ALT levels. **(B)** Plasma AST levels. **(C,D)** Expression levels of pro-fibrogenic genes were examined by qPCR and Western. **(E)** Paraffin sections were stained with picrosirius red and Masson’s trichrome. **(F)** Hepatic hydroxylproline levels. *N* = 8 mice for each group. Error bars represent SD (*p < 0.05, two-way ANOVA with *post-hoc* Scheffe test).

Since HIF-1α appeared to be essential for BRG1 recruitment to the TWIST promoter, we asked whether HIF-1α inhibition would be sufficient to block EndMT and liver fibrosis. We exploited two small-molecule HIF-1α inhibitors, LW-6 (Sato et al., 2015) and YC-1 (Yeo et al., 2003). YC-1 or LW-6 was administered in mice following CCl_4_ injection to evaluate their effects on liver fibrosis. As shown in Figures 5A,B, neither YC-1 nor LW-6 administration resulted in significant changes in plasma ALT and AST levels. In contrast, administration of either HIF-1α inhibitor attenuated CCl_4_-inducd liver fibrosis to a comparable degree as evident in the down-regulation of pro-fibrogenic gene expression levels (Figures 5C,D), reduction of picrosirius red and Masson’s stainings of collagenous tissues (Figure 5E), and decrease in hepatic hydroxylproline quantification (Figure 5F). Combined, these data support a role for HIF-1α in promoting EndMT *in vitro* and liver fibrosis *in vivo*.

### TWIST Inhibition Attenuates Liver Fibrosis in Mice

**FIGURE 6:**
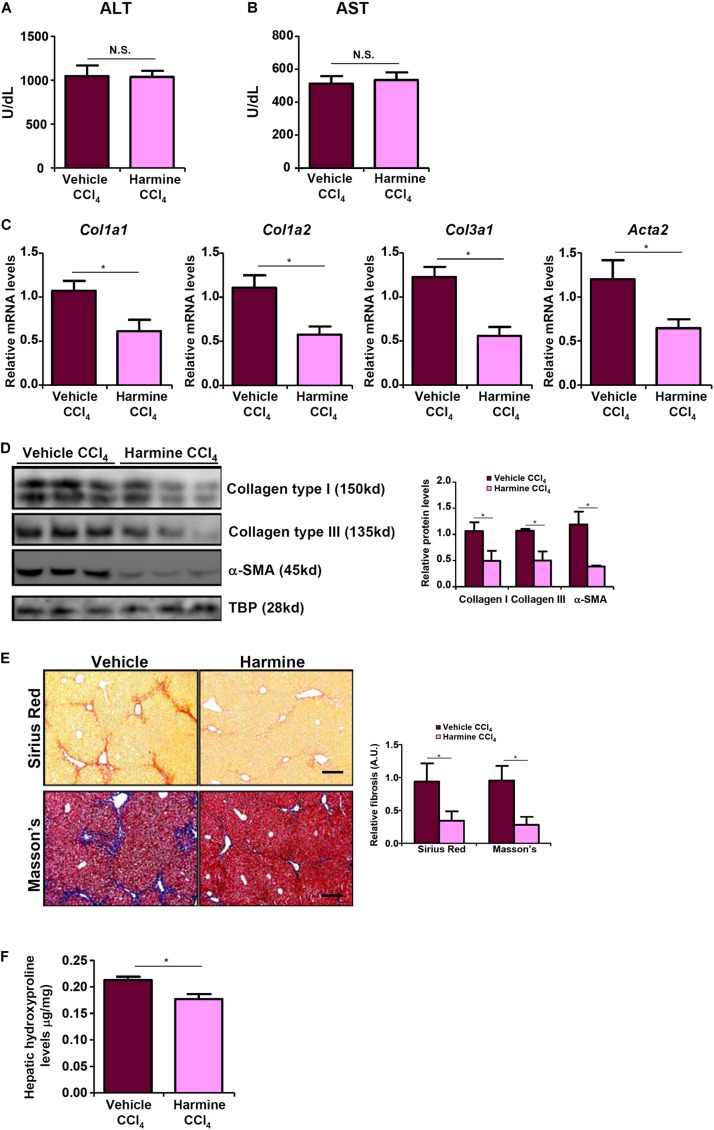
TWIST inhibition attenuates liver fibrosis in mice. Liver fibrosis was induced in by CCl_4_ injection. The mice were injected with the TWIST inhibitor harmine every other day. **(A)** Plasma ALT levels. **(B)** Plasma AST levels. **(C,D)** Expression levels of pro-fibrogenic genes were examined by qPCR and Western. **(E)** Paraffin sections were stained with picrosirius red and Masson’s trichrome. **(F)** Hepatic hydroxylproline levels. *N* = 8 mice for each group. Error bars represent SD (*p < 0.05, two-way ANOVA with *post-hoc* Scheffe test).

Finally we probed the possibility that targeting TWIST with an inhibitor would be sufficient to assuage liver fibrosis *in vivo*. To this end, we injected the mice with harmine, a small-molecule compound that targets TWIST for proteosomal degradation (Yochum et al., 2017). Harmine injection had a marginal influence on CCl_4_-induced liver injury as shown by the minimal changes in plasma ALT (Figure 6A) and AST (Figure 6B) levels compared to the vehicle control. QPCR (Figure 6C) and Western (Figure 6D) analyses revealed that there was a significant reduction in hepatic expression levels of pro-fibrogenic genes as a result of harmine injection as opposed to vehicle injection. Picrosirius red and Masson’s stainings corroborated the finding that TWIST inhibition by harmine led to down-regulation of collagenous tissues in the liver (Figure 6E). Quantification of hepatic hydroxylproline levels confirmed that harmine injection resulted in attenuation of liver fibrosis in mice (Figure 6F).

## Discussion

**FIGURE 7:**
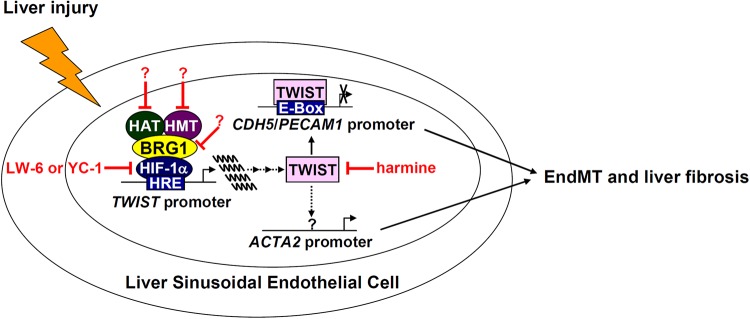
A schematic model. In response to liver injuries, liver sinusoidal endothelial cells undergo EndMT contributing to myofibroblast activation and liver fibrosis. Specifically, HIF-1α recruits BRG1, which in turn recruits histone modifying enzymes (histone acetyltransferase, HAT, and histone methyltransferase, HMT), to the TWIST promoter to activate TWIST transcription. TWIST represses endothelial gene expression and activates mesenchymal gene expression to promote EndMT and liver fibrosis. Pharmaceutical targeting of various factors involved in the proposed model, some of which (harmine for instance) have been tested in this paper, will hopefully provide novel interventional strategies against liver fibrosis.

Activated myofibroblasts that contribute to liver fibrosis are a heterogeneous pool of cells derived from both intra- and extrahepatic sources. Liver sinusoidal endothelial cells (LSECs), by means of EndMT, constitute a small fraction of myofibroblasts in fibrotic livers (Ribera et al., 2017). Here we detail a previously unrecognized role for the chromatin remodeling protein BRG1 in regulating EndMT and liver fibrosis. We demonstrate that BRG1 deficiency alters gene expression patterns reminiscent of EndMT in cell culture and dampens liver fibrosis in mice (Figure 7).

We show here that the ability of BRG1 to regulate TWIST transcription and EndMT in part relies on HIF-1α. Of note, mice with endothelial-specific deletion of HIF-1α appeared normal under physiological conditions (Wei et al., 2012) although it remains to be determined whether these mice would phenocopy the BRG1 ecKO mice with regard to liver fibrosis as reported here. Interestingly, it has been reported that myeloid-derived HIF-1α promotes BDL-induced fibrosis without altering liver injury in mice (Copple et al., 2012). In addition, Mesarwi et al. (2016) have demonstrated that hepatocyte-restricted deletion of HIF-1α attenuates liver fibrosis but minimally influences liver injury in a mouse model of non-alcoholic steatohepatitis. These observations are consistent with the notion that activated myofibroblasts in fibrotic tissues can find their origins in myeloid-derived (Kanisicak et al., 2016) and hepatocyte-derived (Zeisberg et al., 2007) precursors. They also echo our finding that BRG1 deficiency in endothelial cells ameliorates liver fibrosis but not liver injury and raise the possibility that the processes of liver injury and liver fibrosis, though intertwined, can be dissected. BRG1 is known to act as a co-factor for HIF-1α (Sena et al., 2013) but it is unclear whether the behavior of BRG1 in either macrophages or hepatocytes would mirror that of HIF-1α in terms of liver injury. These lingering issues certainly merit additional investigations.

It has become evident that BRG1 can bridge locus-specific histone modifications to transcriptional outcomes. Here we show that BRG1 knockdown skews the chromatin structure surrounding the TWIST promoter region to a more repressive state characterized by lower levels of acetylated H3 and trimethylated H3K4 and higher levels of dimethylated H3K9, which could probably be attributable to altered recruitment of various histone modifying enzymes. The pathophysiological relevance of this finding is open to argument but several recent discoveries offer insights on the subject matter. All three histone modifying enzymes are considered co-factors for HIF-1α tailoring to cellular hypoxic response (Lando et al., 2002; Maejima et al., 2014; Cao et al., 2017). Cells with varying loss-of-function p300 mutations display heightened expression of Tie2, an endothelial cell marker, suggesting that p300 deficiency may pivot cells to an endothelial-like phenotype (Kimbrel et al., 2009). In addition, JMJD1A/KDM3A is a well-characterized transcriptional target of HIF-1α (Beyer et al., 2008). Furthermore, KDM3A activation is found to be associated with a pro-fibrogenic transcriptional program *in vivo* (Zhang et al., 2018a). When pieced together and in combination of the data summarized here, these observations strongly argue for the existence of a large HIF-1α-containing epigenetic complex wherein BRG1 acts as a critical link/node that programs EndMT thereby contributing to liver fibrosis. The composition of this complex and the precise role of each individual component in EndMT and liver fibrosis, however, await further in-depth exploration.

Although we based our model (Figure 7) largely on the role of BRG1 as regulator of EndMT, it should be pointed out that other BRG1-dependent endothelial functions, or lack thereof, may better explain the amelioration of liver fibrosis in the ecKO mice. Endothelial cells contribute to liver fibrosis primarily by regulating hepatic blood flow, by modulating the intrahepatic immune response, by releasing angiocrine signals, and by orchestrating sinusoid capillarization (Poisson et al., 2017; Shetty et al., 2018). For instance, earlier studies have found that BRG1 regulates intravascular leukocyte trafficking by stimulating the transcription of adhesion molecules, including ICAMs, VCAMs, and selectins, in endothelial cells (Chen et al., 2013; Fang et al., 2013; Song et al., 2016; Zhang et al., 2018c). Different immune cell populations exert distinct modulatory effects on liver fibrosis (Xu et al., 2012). It is thus conceivable that there might be a shift in the makeup of the intrahepatic immune-microenvironment, due to differential homing, in the ecKO mice that likely contributes to altered liver fibrosis. Indeed, our recent data suggest that alleviation of unilateral ureteral obstruction induced renal fibrosis in endothelial-specific BRG1 knockout mice could be ascribed to down-regulation of adhesion molecules in the kidneys, which leads to decreased trafficking of macrophages and consequently amelioration of renal inflammation (Liu et al., 2019); a similar scenario may apply the currently investigated experimental setting. BRG1 is also responsible for the synthesis of a host of endothelial-derived bioactive substances, such as endothelin (Yang et al., 2013), NO (Fish et al., 2010), CSF1 (Zhang et al., 2018b), VEGF (Lan et al., 2017), and reactive oxygen species (ROS) (Li Z. et al., 2019), that are implicated in fibrosis. We have recently reported that BRG1 activates the transcription of caveolin-1 (CAV1) in endothelial cells, which in turn represses eNOS activity thus limiting the bioavailability of NO in the liver; deficiency of BRG1 rescues eNOS activity and protects from thioacetamide (TAA) induced liver fibrosis (Shao et al., 2020). Alternatively, we have discovered that BRG1 fuels ROS production in endothelial cells by activating the transcription of NADPH oxidase 4 (NOX4); ROS accumulation in turn drives EndMT to promote liver fibrosis in mice (Li Z. et al., 2019). Recently, Ding et al. (2014) have provided compelling evidence that links endothelial-derived angiocrine signal to liver injury and fibrosis. Specifically, injury-induced angiocrine factors, signaling through the chemokine receptor CXCR4, orchestrate a pro-fibrogenic program in LSECs. CXCR4 is a known transcriptional target for BRG1 during development (Alexander et al., 2015). Therefore, BRG1 deficiency in LSECs may not only curb the production of pro-fibrogenic angiocrine factors but limit the translation of the angiocrine cues to pro-fibrogenic response via signal transduction blockade. This large body of data seems to suggest that multiple inter-related layers of regulatory mechanisms, instead of a single one, may underlie BRG1-dependent transcriptional events in endothelial cells and fibrogenic response in the liver.

Although we showed that the HIF inhibitors (LW-6 and YC-1) and the TWIST1 inhibitor (Harmine) both exerted anti-fibrogenic effects in mice, these observations should be interpreted with caution. First, HIF inhibitors have been previously found to antagonize excessive fibrogenic response in different tissues and organs (Kimura et al., 2008; Qu et al., 2015; Lee et al., 2017). However, it remains undetermined whether these inhibitors specifically target a single population of cells or non-selectively act on several different cell types to achieve the anti-fibrogenic effects. Several independent reports, for instance, have shown that HIF-1α can directly regulate the transcription and phenotype of HSCs (Copple et al., 2011; Wang et al., 2013a; Zhan et al., 2015). Given the wide-ranging roles HIF plays in regulating pathophysiological processes during liver fibrosis (Roth and Copple, 2015), our model that attenuation of liver fibrosis by HIF inhibitors is attributed to modulation of EndMT cannot sustain strict scrutiny and needs to be further examined in EC-specific HIF deletion mice. Second, TWIST-independent effects have been reported for Harmine including activation of inflammasome (Niu et al., 2019), angiogenesis (Dai et al., 2012), and cell proliferation (Dakic et al., 2016). We did not observe any changes in liver injury as judged by plasma ALT/AST levels suggesting that the effect of Harmine on liver fibrosis is unlikely to have occurred secondarily to amelioration of liver injury. Thus, Harmine may preferentially influence one population of cells (e.g., myofibroblasts) over others (e.g., hepatocytes) at least in the current model. However, the notion that this chemical strictly targets TWIST-mediated EndMT is preliminary and awaits further investigation.

In summary, our data show that BRG1 knockdown in cultured endothelial cells blocked pro-fibrogenic stimuli induced down-regulation of endothelial marker genes *in vitro*. BRG1 deletion in endothelial cells attenuated CCl_4_ induced liver fibrosis in mice. The major limitation of the present study is the disconnect between the *in vitro* data and the animal phenotype. Because a previous lineage tracing study has already demonstrated that contribution to the pool of mature α-SMA^+^ myofibroblasts in the fibrotic murine livers by endothelial cells is rather limited (∼5%), our observation that endothelial deletion of BRG1 attenuates liver fibrosis by as much as 50% (depending on the various measurements) simply cannot be explained by the *in vitro* data, which suggest that BRG1 promotes the trans-differentiation of endothelial cells into myofibroblast-like cells. Similarly, the effect of HIF-1α inhibition by small-molecule compound on liver fibrosis is too great to be ascribed to the blockade of endothelial-mesenchymal conversion. The only way to irrefutably argue for a direct role of BRG1-mediated conversion of endothelial cells into myofibroblasts is through lineage fate-mapping, which the current study did not address. As a matter of fact, our data, as they stand, do not support a significant ole for EndMT in contributing to liver fibrosis *in vivo*. Instead, other, much simpler explanations exist that can potentially reconcile the discrepancy between the *in vitro* and the *in vivo* data as presented. Therefore, the impact of our study, from a translational perspective, is rather limited. Additional studies are warranted to clarify the role of endothelial BRG1 in liver injury and fibrosis.

## Data Availability Statement

The datasets generated for this study are available on request to the corresponding author.

## Ethics Statement

The animal study was reviewed and approved by Nanjing Medical University Ethics Committee on Humane Treatment of Experimental Animals.

## Author Contributions

YX and JG conceived the project. WD, MK, YZ, YS, and DW designed experiments, performed experiments, and analyzed data. YX wrote the manuscript. JL and JG secured funding and provided supervision.

## Conflict of Interest

The authors declare that the research was conducted in the absence of any commercial or financial relationships that could be construed as a potential conflict of interest.

## Abbreviations

Brg1: Brahma related gene 1
ChIP: Chromatin immunoprecipitation
EndMT: Endothelial-to-mesenchymal transition
HUVEC: Human umbilical vascular endothelial cell
LSEC: Liver sinusoidal endothelial cells
KDM3A: Lysine demethylase 3A
KDM7: Lysine methyltransferase 7
qPCR: Quantitative polymerase chain reaction
TGF- β: Transforming growth factor beta.
