# Supplementary material for: Activation of TWIST Transcription by Chromatin Remodeling Protein BRG1 Contributes to Liver Fibrosis in Mice
Source: Front Cell Dev Biol. 2020 May 13;8:340. doi: 10.3389/fcell.2020.00340 (PMC7237740; doi:10.3389/fcell.2020.00340)
Supplement: Supplementary file 1 [file Image_1.pdf]

Dong WH et al: Activation of TWIST transcription by chromatin remodeling protein BRG1 contributes to liver fibrosis in mice

Online supplementary material

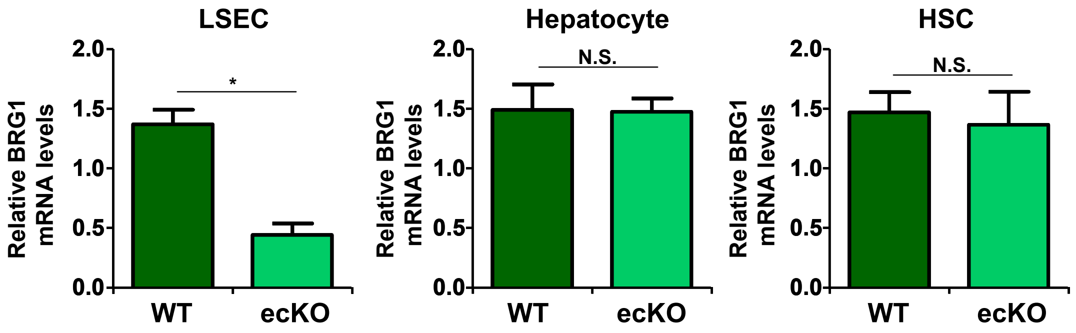

Fig.S1: Primary LSECs, hepatocytes, and hepatic stellate cells were isolated from WT and ecKO mice as described in Methods. BRG1 expression was examined by qPCR. N=3 mice for each group. N.S., no statistical significance.
